# Ecological Drivers and Sex-Based Variation in Body Size and Shape in the Queensland Fruit Fly, *Bactrocera tryoni* (Diptera: Tephritidae)

**DOI:** 10.3390/insects11060390

**Published:** 2020-06-23

**Authors:** Yufei Zhou, Juanita Rodriguez, Nicole Fisher, Renee A. Catullo

**Affiliations:** 1 Department of Ecology & Evolution, Research School of Biology, Australian National University, Acton, ACT 2601, Australia; renee.catullo@gmail.com; 2 Australian National Insect Collection, CSIRO, Acton, ACT 2601, Australia; Juanita.Rodriguez@csiro.au; 3 Digital Collections & Informatics, National Research Collections Australia, CSIRO, Acton, ACT 2601, Australia; Nicole.Fisher@csiro.au

**Keywords:** fluctuating asymmetry, ecological selection, sexual dimorphism, Bergmann’s rule, Allen’s rule

## Abstract

The Queensland fruit fly (*Bactrocera tryoni*; Q-fly) is an Australian endemic horticultural pest species, which has caused enormous economic losses. It has the potential to expand its range to currently Q-fly-free areas and poses a serious threat to the Australian horticultural industry. A large number of studies have investigated the correlation between environmental factors and Q-fly development, reproduction, and expansion. However, it is still not clear how Q-fly morphological traits vary with the environment. Our study focused on three morphological traits (body size, wing shape, and fluctuating asymmetry) in Q-fly samples collected from 1955 to 1965. We assessed how these traits vary by sex, and in response to latitude, environmental variables, and geographic distance. First, we found sexual dimorphism in body size and wing shape, but not in fluctuating asymmetry. Females had a larger body size but shorter and wider wings than males, which may be due to reproductive and/or locomotion differences between females and males. Secondly, the body size of Q-flies varied with latitude, which conforms to Bergmann’s rule. Finally, we found Q-fly wing shape was more closely related to temperature rather than aridity, and low temperature and high aridity may lead to high asymmetry in Q-fly populations.

## 1. Introduction

The Queensland fruit fly (Q-fly), *Bactrocera tryoni* (Froggatt), is a major horticultural pest species in Australia. The Q-fly has a wide range of host species, including hundreds of native and introduced fruits and vegetables [1,2]. Adult Q-flies cause considerable economic losses in Australian horticultural industries as they lay eggs in the fruit of the host species on which the larva feed. It is estimated that the Australian government spent over AUD 48 million on Q-fly control and management during 2006–2009, which included direct control, post-harvest management, surveillance, and research [2]. The Q-fly also negatively impacts fruit and vegetable exports because fruits and vegetables from Q-fly zones need to undergo costly disinfestation procedures prior to export [3].

Before the colonization by Europeans, the Q-fly was native to coastal areas and tropical rainforests of Queensland and northern New South Wales [4,5]. Due to the agricultural expansion of cultivated fruits [4], populations of the Q-fly have expanded southward to Victoria, to some inland areas [2], and have invaded some South Pacific islands [6,7]. Several Q-fly outbreaks have occurred in Western Australia and South Australia, which have a high risk of establishment because the climatic and environmental conditions in these places are suitable for the Q-fly. Through appropriate control and management, the Q-fly has been eradicated in these areas [2]. To reduce the impact of fruit flies in mainland Australia and maintain high-value horticultural markets, the Tri-state Fruit Fly Exclusion Zone (FFEZ) was established in 1994 [7,8]. The FFEZ covers major horticultural farming regions and highly productive areas of south-western New South Wales, north-western Victoria, and south-eastern South Australia [7,8].

Similar to other fruit flies, the survival, reproduction, expansion, and morphometric development of the Q-fly are highly affected by climate, among which, temperature and moisture are the most important factors [2,4]. Temperature is important to Q-fly survival at both extreme low and high temperatures, particularly in immature individuals [7,8,9], as they demonstrate significant developmental stress [10,11]. Moisture and rainfall also play an important role in limiting Q-fly distribution and abundance [4,8,12]. Bateman [12] reported that rainfall deficiency could lead to low fecundity in female Q-fly and high mortality in immature individuals and newly emerged adults, thereby resulting in a Q-fly abundance decrease. While rainfall and moisture could restrict the expansion of the Q-fly, irrigation tends to reduce the effect of low environmental rainfall [4,8], and has allowed the expansion of Q-fly populations into drier areas.Climate change is relatively likely to cause southern fruit fly-free zones to become more suitable for Q-fly colonization [2], and poses a serious threat to the Australian horticultural industry in these areas [2,7,8].

Even though a wide range of studies have examined how environmental factors affect Q-fly distribution and population dynamics, few studies assess their effect on morphological traits like body size, wing shape, and fluctuating asymmetry. Wing length as a proxy for body size, for example, has been found to increase with latitudinal gradient and to be associated with desiccation tolerance in the Q-fly [6]. This is consistent with Bergmann’s rule, which states that individuals along a latitudinal gradient should be larger in higher latitudes as a result of selection for improved thermoregulatory ability [13,14]. It also suggests that body size should be correlated with humidity and precipitation. This correlation has been found for dipterans [15,16,17], where the most common explanation for the body size and desiccation relationship is Allen’s rule (i.e., larger individuals have smaller surface area relative to their volume, protecting them from rapid water loss) [15,18], which therefore increases their survival under desiccation stress. However, other ecological factors, such as diet quality, climatic seasonality, and growing season length, influence insect body size [14,19,20].

Wing shape is also closely associated with environmental factors as a result of increased dispersal and flight ability [21,22,23,24,25] and environmental stress tolerance [10,26,27]. In flying animals, wing shape is measured as a wing aspect ratio (AR), which is the length of the wing relative its width, is a common measurement of wing shape, and is closely related to flight performance in flying animals. AR has been found to be correlated to flight performance in flying vertebrates [28], but this correlation has only been established for few invertebrates groups like anisopteran dragonflies [29], speckled wood butterflies (*Pararge aegeria*) [30], and rain-pool dwelling midges (*Chironomus imicola*) [31], and lower AR individuals tend to disperse a farther distance than their higher AR counterparts. However, insect AR has also been linked to temperature in damselflies [32] and *Drosophila melanogaster* [26].

Fluctuating asymmetry (FA), which refers to deviations away from perfect bilateral symmetry, is often a result of developmental disturbance (i.e., environmental disturbance during development) [11,33], and has been established as a good indicator of environmental stress [7,10,21,34,35]. Since the development of insect wings is controlled by various stress-susceptible genes, relatively minor stress during development has a large effect on wing symmetry [10]. Wings therefore often act as an indicator of environmental stress [10,27]. Field studies, however, indicate that drivers of FA are more complex than previously thought, where other confounding effects in the wild might have an impact on the effect of environmental stress on phenotypic FA [36].

Sexual selection could also lead to trait variation and often leads to sexual dimorphism (two sexes in a species exhibit differences in other morphological characteristics than sexual organs) [37]. In insects, sexual dimorphism is very common, and most insects show morphometric sexual differences in body size, ornamentation, or coloration [37]. In most of the dipteran species, females tend to be larger than males [38,39,40]. This size difference has been explained by (1) a longer development period or faster growth rate in females; (2) increased fecundity in large females [41]; and/or (3) differences in locomotion behaviors, where males are smaller because they require a higher level of flying maneuverability and agility [39]. Wing shape of dipteran species also differs between sexes; females tend to have higher AR wings than males. This is explained by the need for females to fly longer distances [31,39] and have a higher flight efficiency to carry a full load of eggs [31]. In contrast, males often fly shorter distances than females and their flights tend to require greater maneuverability and agility. Therefore, they may be advantaged by having lower AR than females, but faster-beating wings [31].

Of these morphological traits affected by environmental factors, only body size has been studied for the Q-fly [6]. Here, we expand Popa Baez’s [6] study of contemporary Q-fly populations, by investigating a broader range of traits across a well-sampled latitudinal gradient. We measured the morphological trait variation in historical Q-fly samples (collected from 1955 to 1965) to assess whether body is correlated with latitude (Bergman’s Rule) and dryness (Allen’s rule), and whether wing shape and wing symmetry, which are closely related to flight and dispersal ability, are affected by environmental stress in the Q-fly. We also assessed the differences between female and male Q-fly samples in these three traits.

## 2. Materials and Methods

### 2.1. Insect Sampling

**Figure 1:**
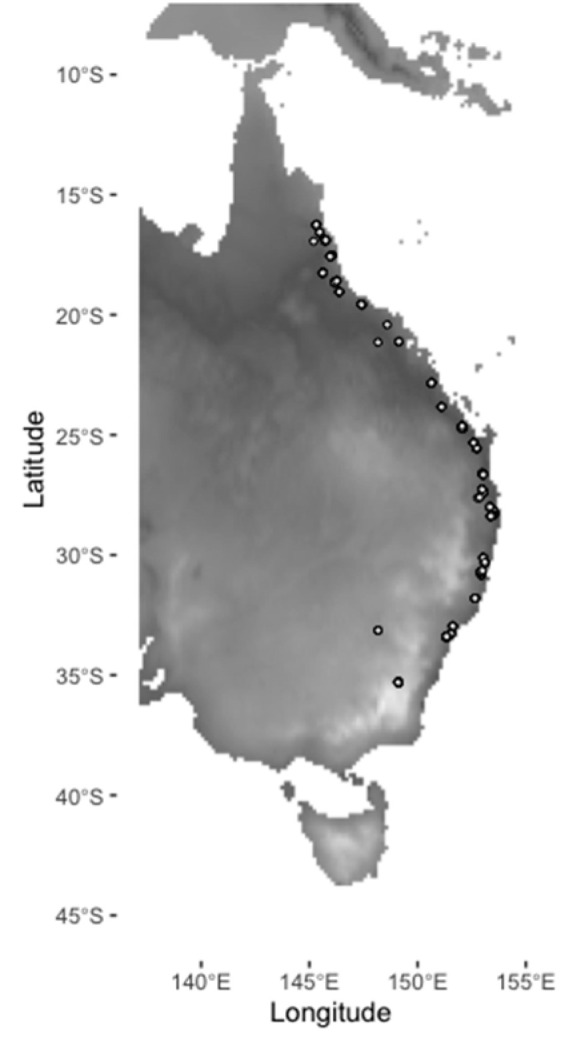
Locations of Queensland fruit fly (Q-fly) specimens examined for morphological variation, mapped over the minimum temperature of the coldest month (Bio06 [1]).

**Figure 2:**
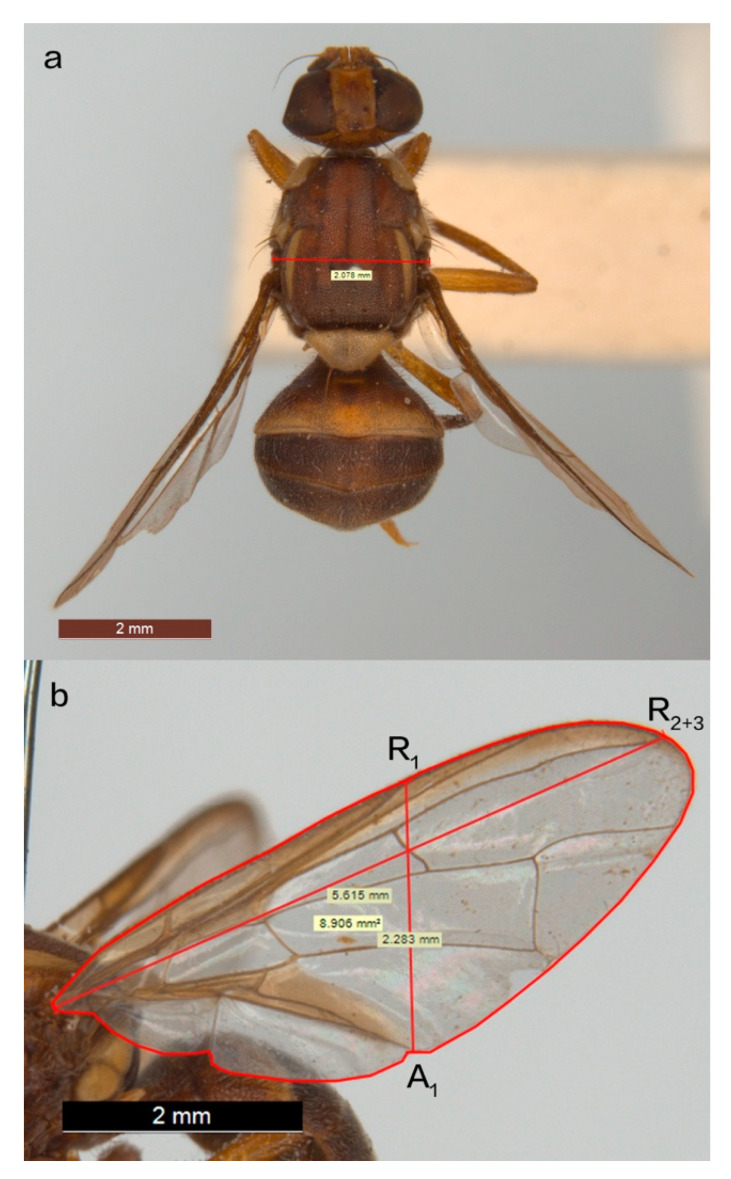
Measurements: (**a**) Intertegular length (a proxy for body size). The red line shows the intertegular length in a Q-fly; (**b**) wing length, width, and area measurements. Wing length was measured from the base of the wing to the end of vein R_2+3_, wing width was measured from the end of vein A_1_ to the end of vein R_1_, and the area of the wing was measured by outlining the edge of the wing.

We imaged a total of 331 individuals, which included 114 males and 217 females (Figure 1). All specimens examined for this research were preserved in the Australian National Insect Collection (ANIC). We chose specimens collected from 1955 to 1965 as this period had the largest sample size across a latitudinal gradient and reduced the possible confounding effects of climate change and agricultural development. During this time period, specimens were collected from 60 sites across a significant latitudinal gradient (−16.25 to −25.334, ~2500 km; Figure 1). For each collection site, we initially examined up to 10 intact specimens (with an undamaged head, thorax, abdomen, and two wings), including 5 females and 5 males. There were 14 sites where the female samples vastly outnumbered the male samples, so we examined more than 5 females at these locations. We examined 138 males and 242 females in total, with females distinguished by the presence of a pointed ovipositor. Specimens were imaged using a Leica M205 C stereo microscope, and trait measurements were made using the Leica application suite. On each individual, we measured the intertegular length (Figure 2a), wing length, width, and area for both wings (Figure 2b). Imaging and measuring works were completed by a single individual (YZ).

We used intertegular length as a proxy for body size, as the curvature of pinned specimens reduces the accuracy of a whole body or whole abdomen approach (Figure 2a). Intertegular length is the distance between the tegulae, a pair of sclerotized structures covering an insect’s wing bases. Intertegular length is a reliable and direct proxy of body size, especially for pinned museum specimens, because it has a positive relationship with body dry weight [42,43]. We also assessed wing shape variation in Q-fly samples using wing aspect ratio (AR). AR is calculated as the wing length divided by wing width [32], using easily identifiable wing structures for standardized measurements. Therefore, wing length (from the base of the wing to the end of vein R_2+3_) and wing width (from the end of vein A_1_ to the end of vein R_1_) were measured for each of the two wings on each individual (Figure 2b). We calculated AR by dividing the average wing length by the average wing width. In order to reduce the effect of allometry [44], we divided wing AR by intertegular length, and we defined this value as relative AR. This allows us to assess whether wing AR varies across ecological gradients independent of body size.

We assessed FA by comparing the area of the left and right wings. Wing area was measured by outlining the edge of the wing for both wings on every individual using the Leica software (Figure 2b). FA was calculated as the area of the larger wing, divided by the area of the smaller wing. During the measurements, all the damaged wings or folded wings were noted, and if identified as outliers, removed from analyses using the wing area.

### 2.2. Climate Data

Previous studies identified temperature, precipitation, and humidity as most influential for Q-fly development and distributional limits [2,7,9,45]. Therefore, we used three variables related to temperature, including annual mean temperature, minimum temperature in the coldest month, and maximum temperature in the hottest month, and four variables associated with rainfall and humidity, including annual mean aridity, precipitation seasonality, precipitation in the driest period, precipitation in the wettest period, and annual mean relative humidity in our analyses. Climatic variables for each individual (Table S1) were downloaded from the Atlas of Living Australia’s spatial portal (https://spatial.ala.org.au). Information on climate variables are in Table 1.

### 2.3. Data Analysis

Statistics were completed in R 3.6.1 [46]. We used the ggpubr [47] and gridExtra [48] packages to visualize our data. The full code is available in the Supplementary Material Table S2.

#### 2.3.1. Trait Variation in Males Versus Females

We used the *t*.test function in the dplyr package [49] to do an unpaired two-sample *t*-test to test for significant differences in trait means between males and females [50]. We identified and re-examined outlier samples, and discarded them if they were (1) juvenile individuals, (2) had damaged or folded wings, or (3) determined it was relatively difficult to confidently image the whole of their wings or tegulae due to the positioning of the preserved specimen.

#### 2.3.2. Investigate the Effect of Environmental Variables on Trait Variation

We used an analysis of covariance (ANCOVA) to test Bergmann’s rule and Allen’s rule in our Q-fly samples. In order to reduce the complexity of each model, we used a stepwise variable selection process (stepAIC), which incorporated all environmental variables, latitude, and sex. We used a generalized linear model (GLM) to test the association between important variables identified in the stepAIC and trait variation in the Q-fly.

An ANCOVA was used to examine differences in the mean values of the response variables that are related to the predictor variables while taking into account the effect of categorical variables [50]. In order to test whether the body size variation in our Q-fly samples conformed to Bergmann’s rule and Allen’s rule, we used the R function anova () to do the ANCOVA to investigate the influence of latitude and aridity on body size variation while considering sex. Meanwhile, in order to test whether the body size variation was related to flight ability in our Q-fly samples, we also used ANCOVA to test the association between body size and AR while considering sex.

StepAIC is a stepwise model selection using the Akaike information criterion (AIC), which simplifies the model without affecting the performance [51]. We used the stepAIC () function in the MASS package [52] on each morphological trait, incorporating all environmental, geographic, and sexual variables, to reduce the complexity of each model. Then, we used GLM on each trait to test the association of the trait with important variables that were retained after stepAIC. GLM is a more flexible extension of the general linear model that allows response variables to have non-normal distribution [53]. We used the glm () function to test the association between trait variation and environmental, geographic, and sexual variables. Regression coefficients (β) indicated the positive (β > 0) or negative (β < 0) relationship between the trait variation and explanatory variables. For fluctuating asymmetry, we used the plot () function to create scatter plots between FA and important environmental variables in order to visualize the distribution of high FA (more asymmetrical) individuals across environmental gradients.

As most previous studies used wing length or size as a proxy of body size [6,15,16,17], we also tested the relationship between intertegular length and wing length, as well as intertegular length and wing area, in order to make our results more comparable to other studies.

## 3. Results

**Figure 3:**
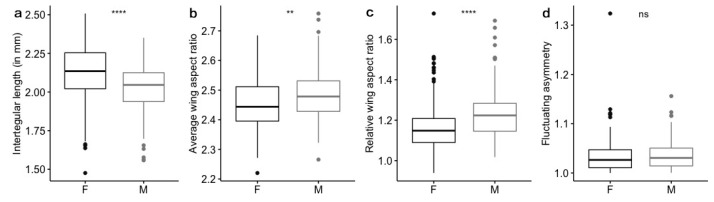
Differences between female (left) and male (right) in (**a**) intertegular length, (**b**) average wing aspect ratio, (**c**) relative wing aspect ratio, and (**d**) fluctuating asymmetry. Two sample *t*-test, ns: *p* > 0.05, **: *p* < 0.01, ****: *p* < 0.0001.

There were significant differences (Figure 3) between males and females in body size (*t* = 5.4727, *df* = 329, *p* < 0.0001), average wing aspect ratio (*t* = −3.2096, *df* = 329, *p* = 0.0015), and relative wing aspect ratio (*t* = −6.1878, *df* = 329, *p* < 0.0001), but we found no significant difference between females and males for fluctuating asymmetry (*t* = 0.23,349, *df* = 329, *p* = 0.8155). Therefore, sex was used as a covariate in the ANCOVA tests for body size and wing shape.

Intertegular length is significantly positively related to wing length (β = 0.332, Std. Error = 0.012, *t* = 28.68, *p* < 0.0001) and wing area (β = 0.106, Std. Error = 0.004, *t* = 26.78, *p* < 0.0001).

### 3.1. Body Size Variation

Intertegular length of females was larger than males (female: *M* = 2.14, *SD* = 0.142 mm; male: *M* = 2.06, *SD* = 0.127 mm). There was a significant relationship between body size and latitude (*χ*^2^ = 19.632, *df* = 1, *p* < 0.0001) and body size and sex (*χ*^2^ = 29.884, *df* = 1, *p* < 0.0001). The interaction term between latitude and sex was non-significant in the ANCOVA. There was no significant relationship between body size and aridity (*χ*^2^ = 1.5091, *df* = 1, *p* = 0.2202), and the interaction term between aridity and sex was non-significant in the ANCOVA. The association between body size and wing shape was also non-significant (*χ*^2^ = 0.9418, *df* = 1, *p* = 0.3325).

A stepwise best subset regression incorporating all climate variables, plus sex and latitude, identified a model that retains sex, annual mean temperature, temperature of the coldest month, and precipitation seasonality to explain body size variation. This model omitted latitude, temperature of the hottest month, precipitation of the driest period, precipitation of the wettest period, annual mean aridity, and humidity as explanatory variables. The GLM on body size (Table 2) incorporating these variables found it was positively associated with minimum temperature in the coldest month (β = 0.017, *p* = 0.0449), and negatively associated with precipitation seasonality (β = 0.002, *p* = 0.0267) and sex (i.e., females were larger; β = 0.090, *p* < 0.0001). Conversely, body size was not significantly associated with annual mean temperature. There were no significant interaction terms between any variables. However, even though stepAIC omitted latitude as an explanatory variable, we also used a GLM on body size and latitude in order to test Bergmann’s rule, and this analysis found a positive association between body size and latitude (β = 0.006, *p* < 0.0001).

### 3.2. Wing Shape Variation

There was a significant relationship between AR and sex (*χ*^2^ = 12.2094, *df* = 1, *p* = 0.0005). There was no significant relationship between AR and aridity (*χ*^2^ = 2.4071, *df* = 1, *p* = 0.1217), and the interaction between sex and aridity was not significant in the ANOVA. A stepwise best subset regression identified a model that retains sex, annual mean temperature, and maximum temperature in the hottest month to explain the average wing AR variation. This model omitted latitude, minimum temperature in the coldest month, annual mean aridity, precipitation in the wettest period, precipitation in the driest period, precipitation seasonality, and humidity as explanatory variables. The GLM on wing AR (Table 2) incorporating these variables found it was positively related to sex (i.e., males have larger wing AR, β = 0.029, *p* = 0.0016) and maximum temperature in the hottest month (β = 0.025, *p* < 0.0001), and negatively related to annual mean temperature (β = 0.013, *p* < 0.0001). There were no significant interaction terms between any variables.

The relationship between relative (body size-corrected) AR and aridity was also non-significant (*χ*^2^ = 0.2428, *df* = 1, *p* = 0.6225), and there was a significant relationship between relative AR and sex (*χ*^2^ = 40.2081, *df* = 1, *p* < 0.0001). The interaction term between sex and aridity was not significant in the ANOVA. A stepwise best subset regression identified a model that retains sex, minimum temperature in the coldest month, maximum temperature in the hottest month, and precipitation seasonality to explain the relative AR variation. This model omitted latitude, annual mean temperature, annual mean aridity, precipitation in the wettest period, precipitation in the driest period, and humidity as explanatory variables. The GLM on relative AR (Table 2) incorporating these variables found that it was negatively related to minimum temperature in the coldest month (β = 0.007, *p* = 0.0084) and was positively associated with sex (i.e., males have larger relative AR, β = 0.063, *p* < 0.0001), maximum temperature in the hottest month (β = 0.014, *p* = 0.0015), and precipitation seasonality (β = 0.001, *p* = 0.0120). There were no significant interaction terms between any variables.

### 3.3. Fluctuating Asymmetry

According to the ANOVA, FA was not significantly associated with sex (*χ*^2^ = 1.4348, *df* = 1, *p* = 0.2319) or aridity (*χ*^2^ = 1.6834, *df* = 1, *p* = 0.1954), and the interaction term between sex and aridity was also non-significant. A stepwise best subset regression identified a model that retains minimum temperature in the coldest month, annual mean aridity, precipitation seasonality, precipitation in the wettest period, and annual mean humidity to explain the FA variation. This model omitted latitude, annual mean temperature, maximum temperature in the hottest month, and precipitation in the driest period as explanatory variables. The GLM on FA (Table 2) incorporating these variables found that it was only positively related to precipitation in the wettest period (β = 0.0006, *p* = 0.0001) and it was negatively associated with minimum temperature in the coldest month (β = 0.003, *p* = 0.0207) and annual mean aridity (β = 0.02, *p* = 0.0475). However, FA was not significantly related to precipitation seasonality and annual mean humidity. There were no significant interaction terms between any variables.

**Figure 4:**
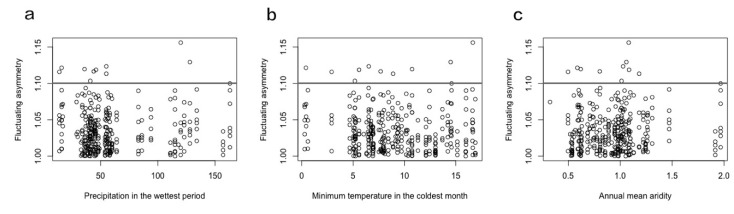
Distributions of Q-fly fluctuating asymmetry on important environmental variables: (**a**) precipitation in the wettest month; (**b**) minimum temperature in the coldest month; (**c**) annual mean aridity. The line shows the high fluctuating asymmetry (FA) value (*M* + 3 *SD*): individuals above the line were defined as highly asymmetric individuals.

In order to see whether those highly asymmetric individuals have effects on the overall trend, we removed individuals with a higher level of FA (individuals with an FA of *M* + 3 *SD*) and conducted stepAIC and GLM again for the retained individuals. The results were similar to the FA of the full data. The stepwise best subset regression identified minimum temperature in the coldest month, annual mean aridity, precipitation seasonality, and precipitation in the wettest period as important variables in explaining the FA variation. The GLM on FA of these lower FA individuals found that precipitation in the wettest period was the only variable that had a significant positive association with FA (β = 0.0004, *p* = 0.0022). Minimum temperature in the coldest month, annual mean aridity, and precipitation seasonality did not play an important role. We also found that most of the individuals with a higher level of FA (greater than *M* + 3 *SD* = 1.10) were found in the locations with moderate environmental conditions of precipitation in the wettest period (Figure 4a), minimum temperature in the coldest month (Figure 4b), and annual mean aridity (Figure 4c).

## 4. Discussion

### 4.1. Body Size Variation

Firstly, we found that sex was the most important variable that was associated with Q-fly body size variation. The body size of female Q-flies was larger than that of males (*t*-test: *p* < 0.0001, Figure 3a). This female-biased body size dimorphism is very common in dipteran species [31,39,40]. The most common explanation is based on the developmental period and growth rate. In other words, females may have a longer developmental period or faster growth rates than males. In our Q-fly samples, there was a bias towards collecting females in March (25% of total female samples), and towards collecting males in April (24% of total male samples). This result may indicate that female Q-fly individuals may have a longer development period or faster growth rate than males. Studies in Caribbean fruit flies (*Anastrepha suspensa*) indicated that growth rate played a more important role than the developmental period in resulting in sexual dimorphism [39,54]. However, no studies have documented developmental rate differences between males and females in the Q-fly, thus further experiments are needed.

Another explanation of the sex-based size differences may be that body size and flight behavior are correlated, where larger body size in females could support longer flight distances while carrying eggs to find breeding sites. In contrast, a smaller body size could provide males with a higher level of flight maneuverability and agility while mating [39]. Some support for this comes from our finding that males have higher AR than females. This could indicate that males may have differences in flight requirements that result in sexual body size dimorphism. Regardless, it is clear that sexual selection affects body size variation in the Q-fly.

Body size also increased towards higher latitudes, which conforms to Bergmann’s rule (Table 2). This result is similar to the pattern identified in contemporary Q-fly samples [6], indicating that there was little detectable change in the relationship between Q-fly body size and latitude during the last ~60 years. Our analysis identified a model with sex, annual mean temperature, minimum temperature of the coldest month, and precipitation seasonality as the retained explanatory variables, although annual mean temperature was not significant (Table 2). Latitude is highly correlated with a variety of climatic variables, and an increasing number of studies showed temperature is not the only and/or most important climatic variable that varies with latitude, including species richness and food availability [55,56,57].

The GLM identified that minimum temperature of the coldest month and precipitation seasonality were important in explaining body size variation in our Q-fly samples (Figure 1 and Table 2). Opposite to expectation, minimum temperature of the coldest month was negatively correlated with body size; in other words, Q-flies were larger in areas with a higher minimum temperature. Clarke et al. [7], O’Loughlin et al. [9], and Yonow and Sutherst [8] all recognized that low temperature would affect mating and development in Q-fly populations, and they indicated that low temperature was the main climatic factor that limited the southward expansion of Q-fly populations. This positive relationship between Q-fly body size and minimum temperature of the coldest month may be because a lower temperature overwhelmingly affects the developmental process of the Q-fly, thereby leading to a smaller body size in areas with a lower minimum temperature.

We found that aridity did not significantly affect the body size of our historical Q-fly samples (*χ*^2^ = 1.5091, *df* = 1, *p* = 0.2202), but precipitation seasonality was negatively related to Q-fly body size (Table 2). According to our finding, Q-flies are larger in areas with lower level precipitation seasonality, i.e., larger in more temperate areas (Table 2). This result was not consistent with Allen’s rule because insects are expected to enhance their desiccation tolerance by getting larger, which reduces their surface area relative to volume, thereby protecting them from rapid water loss [15,16,17,58]. We hypothesize that our finding of a negative association between body size and precipitation seasonality might be explained by the impact of precipitation seasonality in Q-fly food sources and abundance [14]. Diet quality could have a higher impact on larval stages as it has been demonstrated that alimentary and nutritional quality at the larval stage could affect the insect developmental rate, as well as adult body size [59] and reproductive performance [60]. Adult Q-flies feed mainly on an unidentified bacteria [7], and there is no information on how precipitation impacts on the abundance of these bacteria.

Overall, consistent with other studies, our Q-fly samples have obvious female-biased body size dimorphism, and our historical Q-fly samples conform to Bergmann’s rule. This appears to be driven by minimum temperatures versus annual mean temperatures or highest temperatures. However, opposite to previous studies, we found that our Q-fly samples do not conform to Allen’s rule, and precipitation seasonality is more important than aridity in explaining the Q-fly body size variation.

### 4.2. Wing Shape Variation

We found a significant difference between female and male Q-fly vouchers in wing shape. Both the average AR value (*t*-test: *p* = 0.0015, Figure 3b) and relative AR (*t*-test: *p* < 0.0001, Figure 3c) of males were higher than that of females, indicating that males had longer and more slender wings than females even when controlling for body size differences between the sexes. This result does not correspond with a wide range of studies based on dipteran species showing that females generally have longer wings because they need higher flight efficiency to fly longer distances while carrying eggs [31]. One possible explanation is that Q-flies are more sedentary when the fruit is available and abundant, and this may have a stronger effect in adult females because host fruits are important for females to lay eggs [61,62,63]. We found that male AR was not related to any environmental variables tested in our study, but increased moisture was correlated with female Q-flies having shorter and wider wings (Figure 3c). These results are concordant with our hypothesis that female AR might be affected by increased host abundance where there is higher precipitation in the driest period and lower precipitation seasonality.

Longer wings in males could also be related to mate-seeking behavior. Q-fly males are strongly attracted to a specific scent, a phenomenon known as “cue-lure” [64]. Due to this phenomenon, male Q-flies are more likely to fly longer distance than females to seek for a chance of mating [7,61]. According to Dalby-Ball and Meats [61], the presence of cue-lure may increase male flight activity, but female Q-flies showed little response to cue-lure [7]. Thus, male Q-fly samples may have a higher AR because they have more flight-intensive mate-seeking behaviors.

Both AR and relative AR variations were closely related to temperature in our Q-fly samples. One unique pattern we found was that both AR and relative AR were positively correlated with maximum temperature of the hottest month (Table 2). However, AR was negatively associated with annual mean temperature and relative AR was negatively related to minimum temperature of the coldest month (Figure 1). This pattern might be the result of wing shape being sensitive to environmental pressure [10,27]. The relationship between temperature and Q-fly survival has been widely studied [9]. An annual mean minimum temperature lower than 2.6 °C results in an extremely low adult winter survival rate in Q-fly populations, and some adults survive with a daily maximum temperature of ~38–40 °C [7]. The influence of temperature varies with the Q-fly life stage; immature individuals are more vulnerable to extreme temperatures [7,9], which can lead to developmental stress [10,11]. The close relationship between wing shape and temperature was also found in damselflies [32] and *Drosophila melanogaster* [26]. These two studies revealed the effect of lower temperature on wing shape, indicating that high AR is required in colder areas because higher AR is related to high flight efficiency. Therefore, the pattern of longer wings associated with colder temperatures in the Q-fly may be due to increased flight efficiency under stressful environmental conditions.

Overall, opposite to other studies, we found that male Q-flies have longer and more slender wings than females. We speculate that this pattern might be related to host abundance or the cue-lure phenomenon in male Q-flies. One relative novel finding was that wing shape of the Q-fly is closely correlated with temperature extremes. This result indicated that both low temperature and high temperature could act as environmental stresses on Q-fly populations.

### 4.3. Fluctuating Asymmetry Variation

We found that females and males did not vary in FA. Although females and males may show different responses to environmental stress [65], this was not detectable in our analysis.

Our stepAIC and GLM analyses (Table 2) found minimum temperature of the coldest month and annual mean aridity were negatively associated with FA variation. This result indicated that low temperature (Figure 1) and high aridity could lead to higher asymmetry in Q-fly populations. This finding was similar to the findings of Clarke [66], Dominiak et al. [4], O’Loughlin et al. [9], and Yonow and Sutherst [8], which identified low temperature and dry stress as the key factors that could restrict the distribution of abundance of the Q-fly. Here, we found that these two environmental stresses could not only affect the distribution and abundance of Q-fly populations, as found in previous studies, but also development.

One unexpected result was that precipitation in the wettest period was positively related to the FA variation, which means that Q-flies are more asymmetrical in areas with higher precipitation in the wettest period. After removing outliers (FA > *M* + 3 *SD* individuals, Table 2), we again found that precipitation in the wettest period was positively related to FA. Most studies indicate that higher levels of precipitation and moist environments are important for Q-fly survival, especially for their pupal stage because their pupation occurs in soil [4,12]. Thus, this finding is the opposite of our expectation. The relationship between precipitation in the wettest period and Q-fly FA could be explained by the possibility that areas with the greatest precipitation are not highly suited for Q-fly development. Q-fly pupal mortality is known to be relatively high at extremely high and low soil moistures [67].

Meanwhile, we also found an intriguing pattern where highly asymmetric individuals (FA > *M* + 3 *SD* individuals) were located in areas with a moderate level of climatic conditions (Figure 4). This result was not expected because we hypothesized that the most asymmetric individuals would be more likely in locations with the harshest environmental conditions. One possibility was that locations with moderate environmental conditions may have higher levels of horticultural farming, so the pesticide application might be more intensive, which is known to affect wing size symmetry [10]. The use of pesticides in these moderate climate regions might be a reason for a higher FA level. Therefore, we might be able to use FA to monitor the level of stress imposed by pesticide use. This finding suggests research into how pesticides affect Q-fly development, and downstream fecundity may be valuable to models of Q-fly population dynamics.

Overall, as expected, low temperature and high aridity are correlated with higher morphological asymmetry, suggesting important unknowns are affecting Q-fly development. We also found that those highly asymmetric Q-fly individuals were collected in areas with temperate environmental conditions, and suggest this might be related to more intensive horticultural farming and pesticide applications in these areas.

## 5. Conclusions

We found sexual dimorphism in body size and wing shape, but similar symmetrical growth in the Q-fly. Therefore, drivers such as sexual selection and differing ecological requirements may play an important role in the selection of body size and wing shape between sexes. We also found that our historical Q-fly samples conformed to Bergmann’s rule, and this might be related to temperature change along the latitudinal gradient. We found minimum temperature in the coldest month was significantly related to body size and wing shape as well as fluctuating asymmetry variations, identifying low temperature as a key environmental variable affecting the morphological traits of the Q-fly. However, our Q-fly samples did not conform to Allen’s rule, as Q-fly body size and wing shape might be more sensitive to temperature change and climatic instability. We found the Q-fly was more asymmetrical in areas with moderate environmental conditions, this pattern might be related to intensive farming and pesticide use. Understanding ecological and sexual drivers of body size and shape variation in Q-fly enables us to better monitor and predict Q-fly population dynamics. We suggest that more research on Q-fly morphological traits variation based on contemporary samples is necessary to assess how patterns of trait variation and stress-associated asymmetry have changed through time.

## Figures and Tables

**Table 1:** Environmental data used in this study, as downloaded from the Atlas of Living Australia (ALA).

Variable	ALA Layer Name [Layer ID]
Annual mean temperature	Temperature—annual mean (Bio01) (874)
Minimum temperature in the coldest month	Temperature—coldest month min (730)
Maximum temperature in the hottest month	Temperature—hottest month max (729)
Annual mean aridity	Aridity index—annual mean (715)
Precipitation seasonality	Precipitation—annual seasonality (772)
Precipitation in the driest period	Precipitation—driest period (Bio14) (872)
Precipitation in the wettest period	Precipitation—wettest period (Bio13) (866)
Annual mean relative humidity	Humidity—annual mean relative (728)

**Table 2:** Generalized linear model (Gaussian distribution) regression of relationships between Q-fly traits and environmental variables. Significant relationships between traits and variables are highlighted in bold.

***Body Size***				
**Explanatory Variable**	**β (Regression Coefficient)**	**Standard Error**	**95% Confidence Interval**	***p***
Sex	−0.090	0.015	−0.12, −0.06	**<0.0001**
Annual mean temperature	−0.022	0.011	−0.04, 0.001	0.0595
Minimum temperature of the coldest month	0.017	0.008	0.0005, 0.03	**<0.0001**
Precipitation seasonality	−0.002	0.001	−0.003, −0.0002	**0.0267**
Latitude	0.006	0.001	0.003, 0.008	**<0.0001**
***Wing Shape (AR)***				
**Explanatory Variable**	**β (Regression Coefficient)**	**Standard Error**	**95% Confidence Interval**	***p***
Sex	0.029	0.009	0.01,0.05	**0.0016**
Annual mean temperature	−0.013	0.003	−0.02, −0.007	**<0.0001**
Maximum temperature in the hottest month	0.025	0.005	0.02, 0.03	**<0.0001**
***Wing Shape (Relative AR)***				
**Explanatory Variable**	**β (Regression Coefficient)**	**Standard Error**	**95% Confidence Interval**	***p***
Sex	0.063	0.009	0.04, 0.08	**<0.0001**
Minimum temperature in the coldest month	−0.007	0.003	−0.01, −0.002	**0.0084**
Maximum temperature in the hottest month	0.014	0.005	0.006, 0.02	**0.0015**
Precipitation seasonality	0.001	0.0004	0.0002, 0.002	**0.0120**
***Fluctuating Asymmetry***				
**Explanatory Variable**	**β (Regression Coefficient)**	**Standard Error**	**95% Confidence Interval**	***p***
Minimum temperature in the coldest month	−0.003	0.001	−0.005, −3.95 × 10^−4^	**0.0207**
Annual mean aridity	−0.023	0.012	−0.046, −3.48 × 10^−4^	**0.0475**
Precipitation seasonality	−0.0004	0.0002	−0.0007, 8.63 × 10^−6^	0.0560
Precipitation in the wettest period	0.0006	0.0002	0.0003, 9.61 × 10^−4^	**0.0001**
Annual mean humidity	0.002	0.001	−0.004, 4.63 × 10^−4^	0.1265
***Fluctuating Asymmetry (FA < M + 3 SD Subset)***				
**Explanatory Variable**	**β (Regression Coefficient)**	**Standard Error**	**95% Confidence Interval**	***p***
Minimum temperature in the coldest month	−0.002	0.001	−0.003, 4.47 × 10^−5^	0.0573
Annual mean aridity	−0.017	0.010	−0.037, 2.27 × 10^−3^	0.0838
Precipitation seasonality	−0.0003	0.0006	−0.0007, 6.37 × 10^−5^	0.1101
Precipitation in the wettest period	0.0004	0.0002	0.0003, 7.04 × 10^−4^	**0.0022**

## Supplementary Materials

The following are available online at https://www.mdpi.com/2075-4450/11/6/390/s1, Table S1: *Bactrocera tryoni* individual data; Table S2: R code script for data analysis.
